# Who victimizes whom and who defends whom? A multivariate social network analysis of victimization, aggression, and defending in early childhood

**DOI:** 10.1002/ab.21760

**Published:** 2018-03-25

**Authors:** Gijs Huitsing, Claire P. Monks

**Affiliations:** ^1^ University of Groningen Groningen the Netherlands; ^2^ University of Greenwich London United Kingdom

**Keywords:** aggression, defending, early childhood, social networks, victimization

## Abstract

The aim of this research was to investigate the interplay between victim‐aggressor relationships and defending relationships in early childhood to test the proposition that young aggressors are less selective than older children in their choice of vulnerable targets. Cross‐sectional multivariate statistical social network analyses (Exponential Random Graph Models) for a sample of 177 preschoolers from seven classes, 5‐ to 7‐years‐old, revealed that boys were more aggressive than girls, toward both boys and girls, whereas defending relationships were most often same‐sex. There was significant reciprocity in aggression, indicating that it was more often bidirectional rather than unidirectional. In addition, aggressors clearly defended each other when they shared their targets of aggression, whereas a marginally significant trend appeared for defending between victims who were victimized by the same aggressors. Furthermore, teacher‐rated dominance was positively associated with children's involvement in both aggression and victimization, and teacher‐rated insecurity was associated with less aggression, but not with victimization. These findings suggest that those who are reported as being victimized may retaliate, or be aggressive themselves, and do not display some of the vulnerabilities reported among older groups of victims. The findings are in line with the proposition that young aggressors are less strategic than older children in targeting vulnerable victims. The network approach to peer victimization and defending contributes to understanding the social processes facilitating the development of aggression in early childhood.

## 1 INTRODUCTION

A large proportion of children in Western countries start preschool or school between the ages of 3 and 6 years, and peer victimization can be observed among children of this age (Saracho, 2016; Vlachou, Andreou, Botsoglou, & Didaskalou, 2011), although research is still limited in comparison to the volume of studies published on peer victimization among older children and adolescents (see Smith, 2011). Because aggression at an early age increases the risk of future aggression (Barker et al., 2008), it is imperative to recognize aggressive behavior early. It is therefore important that research on peer victimization should focus on the point at which children first begin preschool or school.

The current study employed a social network approach during the first years of schooling in the United Kingdom (aged 5–7 years) to investigate the relationships between aggressors and the specific targets of their aggression, and between defenders and the children they defend. Aggression, victimization, and defending are specific forms of relational behavior that are embedded in larger group processes, and these relationships are usually investigated from middle childhood into adolescence (e.g., Salmivalli, 2010). It is our aim to investigate the interplay between victimization/aggression (“who is victimized by whom”) and defending (“who defends whom”) relationships in early childhood. This allows us to examine how young children form relationships, which contributes to understanding the early emergence of aggressive behavior in social processes.

### 1.1 Victimization, aggression, and defending in early childhood

Behavior such as aggression and defending can be viewed as occurring in the pursuit of gaining and maintaining status/dominance and affection (Veenstra et al., 2010). Aggression can be used to satisfy status needs (Hawley & Geldhof, 2012; Sijtsema, Veenstra, Lindenberg, & Salmivalli, 2009), but this can be at the expense of affectional ties to peers (Veenstra et al., 2013). In contrast, defending classmates can be seen as a way to obtain affection, as defending is often seen between children who like each other (Sainio et al., 2011). Aggressors are likely to be strategically selective in whom they target in order to enhance their status while maintaining the affection of their preferred group. This means that aggressors will target peers who have a low standing in the peer group. However, contrary to late childhood and adolescence, young children may not be as selective when choosing peers as targets of their aggression. Young children rely more on parents and other adults to meet affectional needs, and may be less dependent on the affection of the peer group (e.g., Von Salish, 2001). Young aggressors may also be less skilled than older children in identifying those who will less likely retaliate, and less stable group processes suggest that victimization is more often a transient experience (Camodeca et al., 2015, Hanish & Guerra, 2000; Perry, Perry, & Boldizar, 1990). Several hypotheses can be derived to test whether young aggressors are less selective in their choice of suitable targets.

First, aggression is likely to be more stable than victimization in early childhood. The role of being an aggressor and the form of aggression show considerable stability (Ladd & Burgess, 1999; Monks et al., 2003; Murray‐Close & Ostrov, 2009; Ostrov, 2008). However, if aggressive children are less selective in targeting peers, they are likely to change targets more frequently, so that reports of victimization (being the target of aggression) would indicate lower stability. Indeed, victimization tends to be short‐lived for many children during early childhood (Kochenderfer & Ladd, 1996; Kochenderfer‐Ladd & Wardrop, 2001; Monks et al., 2003; Snyder et al., 2003). This implies that less selective aggressors may target children who are able to stand up for themselves. These victims may retaliate to aggression (Hanish, Sallquist, DiDonato, Fabes, & Martin, 2012). As a consequence, we predicted reciprocal aggression in early childhood (*H1*).

**Figure 1:**
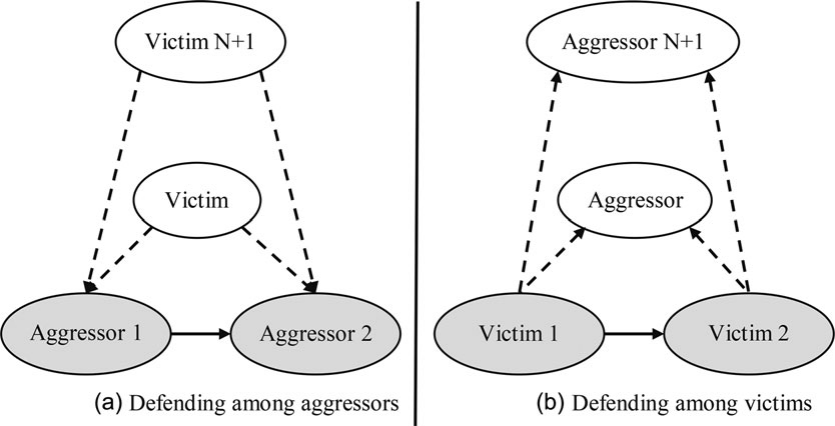
(a) Defending among aggressors. (b) Defending among victims. Dotted lines indicate victim‐aggressor relations, in which the tie is drawn from the victim (sender) to the aggressor (receiver). Solid lines indicate defending relations, in which the tie is drawn from the victim (sender) to the defender (receiver)

Second, supporting relations are expected to be less likely between victims than between aggressors. Aggressors may defend each other when they target the same victims to satisfy affection needs and to prevent retaliation, a pattern that is shown in Figure 1a (Huitsing et al., 2014; Huitsing & Veenstra, 2012). Also, victims who are being victimized by the same aggressors may see defending against their aggressors as a way to satisfy affection needs (see Figure 1b). If aggression is more stable and visible than victimization, aggressors will be able to identify other aggressors more easily than victims would. Thus, we predicted (*H2*) that defending among aggressors (when targeting the same victims, see Figure 1a) would be more likely than defending among victims (when being targeted by the same aggressors, see Figure 1b).

Third, if young aggressors are less selective in their choice of victims, they may not necessarily target the most vulnerable peers. Thus, although there is some indication that aggression (in particular when accompanied by prosocial behavior) is associated with dominance in early childhood (Hawley & Geldhof, 2012; Pellegrini et al., 2007; Perren & Alsaker, 2006), victims are expected to have similar levels of dominance as other children and are not expected to exhibit vulnerabilities such as higher levels of insecurity. Previous research indicated that young victims indeed did not show some of the vulnerabilities identified among older victims such as attachment insecurity, poorer social cognitive skills, or lower sociometric status (Monks et al., 2011; Monks, Smith, & Swettenham, 2005). However, other studies have documented that young victims are less dominant and assertive than others (Perren & Alsaker, 2006; Schwartz, Dodge, & Coie, 1993). Thus, we predicted that aggressors are more dominant and less insecure than non‐aggressors (*H3*) and that victims would not differ in dominance or insecurity from non‐victims (*H4*). Moreover, reciprocal aggression between aggressors who are equally strong can be seen as competition for a place in the hierarchy. Therefore, we predicted that victims would not differ in dominance or insecurity from their specific aggressors (*H5*).

Furthermore, we also considered the role of sex, as it is an important characteristic that influences selectivity in children's relationships. Given that young children's play and friendship groups are often sex‐segregated, it is likely that younger boys and girls may be particularly likely to exhibit same‐sex rather than cross‐sex directed behavior; both positive and negative, as a reflection of the higher levels of contact that they have with same‐sex peers (Fabes, Martin, & Hanish, 2003; Mehta & Strough, 2009). In early childhood, boys are more likely than girls to be aggressors (Camodeca et al., 2015; Kirves & Sajaniemi, 2012; Veenstra et al., 2013; Verlinden et al., 2014; von Grünigen, Perren, Nagele, & Alsaker, 2010) whereas girls are more likely than boys to be defenders (Belacchi & Farina, 2010; Lee, Smith, & Monks, 2016; Monks et al., 2011, 2003). Sex differences in victimization are, however, less consistent (e.g., Lee et al., 2016; Monks & Smith, 2010; von Grünigen et al., 2010). Taken together, we predicted that boys would be more likely to be identified as aggressors (*H6a*) and girls would be more likely to be identified as defenders (*H6b*). We further predicted that same‐sex aggression (*H7a*) and same‐sex defending (*H7b*) would be more common than cross‐sex aggression and defending, respectively.

### 1.2 Current study

In this study, we employed social network analysis in early childhood to test our hypotheses by investigating simultaneously “who victimizes whom?” (Rodkin & Berger, 2008; Veenstra et al., 2007) with the relationships between defenders and the children they defend (Sainio et al., 2011). Social network analysis contributes to understanding aggression, victimization, and defending in the larger social context by accounting for the interdependent nature of these relationships. Social network studies on victimization and defending have tended to focus on the behavior of children in middle/late childhood and preadolescence. The current study aimed to extend this research to examine social processes in peer victimization among 5‐ to 7‐years‐old. We investigated the interplay between victimization and defending relationships within the same classroom, and accounted for child characteristics of teacher‐based levels of dominance and insecurity and sex.

## 2 METHOD

### 2.1 Participants

A total of 200 children (54.5% boys, *N* = 109) and their teachers participated in the study in 2014. The children were aged five to seven years (*M* age 75.6 months; *SD* = 10.39), from eight classes (Reception, aged 4 to 5 years; Year 1, aged 5 to 6 years; and Year 2, aged 6 to 7 years) in three primary schools in the south east of England. Class sizes ranged from 21 to 29. One Reception class (23 children, *M* age 68 months, 13 boys) was excluded from further analyses because the number of defending nominations was exceptionally high (93% of possible relations were mentioned), leaving no variation in the defending network. This resulted in a final sample of 177 preschoolers in seven classes.

### 2.2 Procedure

Ethical approval was obtained from the University Research Ethics Committee. Consent for participation was obtained from headteachers, parents/carers, and class teachers. No parents/carers refused participation. Children were informed that they did not have to take part in the study, that they could withdraw at any time and that responses were confidential. When children reported being victimized, they were encouraged to tell someone and the researcher offered help in addressing the teacher. Trained researchers (*N* = 5, all female) conducted the interviews with each child individually in a designated quiet area within the school. Each interview took approximately 20 min and each child was given a sticker for their participation. Teachers were given the questionnaires to complete in their own time.

### 2.3 Measures

#### 2.3.1 Victim‐aggressor and victim‐defender networks

An interview technique using cartoon prompts was adapted from Monks et al. (2003). Each child was shown four stick‐figure cartoons depicting four different types of aggression: physical (hitting, kicking or pushing another); verbal (shouting at or saying nasty things to another); direct relational (telling another that they cannot join in); and indirect relational (spreading nasty stories about another). The child was shown one cartoon at a time and asked to identify the situation, which was then confirmed for them. The child was asked to identify anyone in their class who behaved aggressively in this way and to whom. The four forms of victimization‐aggression relationships (physical, verbal, direct, and indirect relational) were combined into a measure of general aggression. Thus, when a victim‐aggression relationship was mentioned at least once, we considered this relationship to be present. There are good reasons for combining aggression types, because there is a strong conceptual overlap between the aggression types. We aimed to investigate general aggression, regardless of different processes for different types of aggression. Moreover, analyzing the types of aggression separately would lead to very sparse networks that cannot be estimated with our networks models.

Once victim‐aggressor relationships were identified, children were reminded who they had identified as victims and were asked to report on who defended each victim, resulting in the identification of victim‐defender relationships. This was repeated for each form of aggression. In this way victim‐defender ties were obtained. All relationships were coded such that the victim is the sender and the aggressor or defender the receiver of a tie. Ties were included if at least one peer reported the victim‐aggressor or victim‐defender dyad.

#### 2.3.2 Teacher questionnaire

Items adapted from the Reactive Proactive Aggression Questionnaire for Teaching Staff by Dodge and Coie (1987) were used to ask teachers about children's dominance and insecurity. Teachers completed a questionnaire about each participating child from their class. Teachers rated the statements using a Likert scale from 1 to 7, “1 = never” to “7 = almost always.” The questionnaire measured four dimensions, but only dominance (five items, e.g., “This child usually wants to be in charge or sets the rules and gives orders”, α = .87) and insecurity (six items, e.g., “This child is anxious and insecure in social situations, α = 0.87) are reported here.

### 2.4 Analytical strategy

The networks were analyzed using Exponential Random Graph Models (ERGMs, see, e.g., Lusher et al., 2013), which were estimated using the program *XPNet* (Wang, Robins, & Pattison, 2009). The ERGMs predict the presence of a relationship in a network from several predictor variables, for which parameters are estimated and specified in the model. Combining parameter estimates leads to the interpretation of network formation processes in the observed network. The parameters used in this study were chosen because they lead to a good estimation of the network structures of positive (defending), negative (victimization), and combined positive‐negative networks (Huitsing et al., 2012).

Four models were estimated. Model 1 included structural network parameters that capture the structure of the victimization and defending networks separately and the interplay between these networks. Parameters were specified at the dyadic (relational: between two persons), triadic (involving three persons), and higher‐order (more than three persons) level. These structural parameters model reciprocity in aggression (H1) and defending among bullies and victims (H2). In Model 2, effects for dominance and insecurity were estimated. We considered sender and receiver effects to examine whether child characteristics were associated with aggression and defending others (*receiver* effects for victimization and defending networks, respectively—test of H3) and victimization and being defended (*sender* effects—test of H4). The *absolute difference effect* was included to examine whether differences between victims and aggressors/defenders in dominance/insecurity had an additional effect on the presence of victimization and defending relations, above the sender and receiver effects, which is used to test H5. Model 3 presents results for sex, which was included as a relational covariate to test H6 and H7. With Girl‐Girl relations as the reference category, we examined whether Boy‐Boy, Girl‐Boy (i.e., girl victimized/defended by a boy) and Boy‐Girl relations were more or less likely. In Model 4, all effects were included simultaneously to investigate their relative strength. The number of network relations was fixed in all models because this improves model convergence considerably (Lubbers & Snijders, 2007).

The models for the seven separate classrooms were combined using the meta‐analytic procedure described in Lubbers and Snijders (2007). The obtained estimated mean parameter represents an unstandardized aggregated estimate across classrooms (along with its standard error); the accompanying standard deviation represents the degree to which estimates vary across classrooms. The statistical significance of the mean parameters was tested by dividing the estimate by its standard error; this ratio was tested using a *t*‐ratio, which has approximately a normal distribution. The significance of the parameters for the standard deviations was tested using a chi‐square difference test with 1 degree of freedom.

## 3 RESULTS

### 3.1 Descriptives

Descriptive statistics for the victimization and defending networks are given in Table 1, separated for the relational (network), individual (child), and classroom level. At the relational level, the prevalence of defending and victimization were comparable; about thirty percent of the possible relations were present. Boys were more often aggressors than girls, both toward boys (38%) and girls (35%), with girls aggressing more to girls (21%) than to boys (16%). Boys and girls were equally likely to be victimized. Defending appeared more often same‐sex than cross‐sex. Of the possible relations among girls, almost fifty percent were reported as defending relations.

**Table 1:** Descriptive statistics for the full sample (*N* = 177, 7 schools)

	Victimization	Defending
**Relationship/network level**		
Prevalence (density)^a^	1,238 (28%)	1,270 (29%)
Sex composition^b^		
Girl‐girl	182 (21%)	391 (45%)
Boy‐girl	179 (16%)	251 (23%)
Girl‐boy	383 (35%)	201 (18%)
Boy‐boy	494 (38%)	427 (33%)
**Individual/child level**		
Average in/outdegree	6.99	7.18
Standard deviation outdegree (given nominations)	3.36	5.50
Standard deviation indegree (received nominations)	7.26	4.80
**Classroom level**		
Number of students, of which:	177	177
Number of pure aggressors/defenders	0	6
Number of pure victims	29	9
Number of aggressor‐victims/victim‐defenders	148	154
Number of isolates (non‐involved)	0	8
Reciprocity over all classrooms (*standard deviation*)	38% (10%)	46% (19%)

^a^ The density is the number of relations, relative to the total number of possible relations (4,356).

^b^ The first person is the sender, the second person is the receiver of a relation (i.e., girl‐boy means that a girl is victimized by a boy). The percentages are relative to the total number of possible sex‐relations, which are: girl‐girl = 866; boy‐girl = girl‐boy = 1,091; boy‐boy = 1,308.

At the individual level, it was reported that children were on average victimized by seven peers (average in/outdegree), where they had also on average seven defenders. The standard deviation was larger for the number of received (aggressor) nominations than for the number of given (victim) nominations, suggesting that the differences between children were larger for aggression than for victimization.

The descriptives at the classroom level show that victimization and defending were common phenomena in preschools. All children were involved in at least one victimization or aggression tie (either as pure victims, or as aggressor‐victims), and only eight children were not involved in defending (so‐called “isolates”). There were somewhat more reciprocal nominations in the defending networks (46%) than in the victimization networks (38%).

### 3.2 Network analyses

Table 2 provides the results for the network model with structural network parameters. The first part contains the effects for the victimization network. Victimization is characterized by *reciprocity* (parameter #1 in Table 2 *; Parameter Estimate[P.E])* *= *0.58, *p *< .01), suggesting a tendency of mutual aggression (in line with H1). The positively estimated *in‐ties spread* (#2; *P.E. = *0.64, *p *< .01) means that the distribution of the received aggression nominations was dispersed; some children were more frequently nominated as aggressors than others. The *shared in‐ties* (#4) and *shared out‐ties* (#5) were included in the model because they are partly contained in the multivariate parameters (parameters #14 and #15, see final part of Table 2) and were required to counterbalance the multivariate effects.

**Table 2:** Multivariate network models (ERGMs) for victimization and defending

		Model 1: structural parameters			
		Mean parameter		Standard deviation	
Parameter	Graphical representation	PE	SE	Est.	*χ* ^2^
**Victimization**					
1. Reciprocity	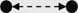	0.58	(0.16)**	0.12	‐0.07
2. In‐ties spread	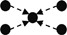	0.64	(0.22)**	0.00	0.00
3. Multiple two‐paths	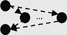	−0.04	(0.03)	0.06	0.41
4. Shared in‐ties	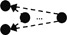	−0.17	(0.08)*	0.00	0.00
5. Shared out‐ties	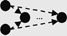	0.27	(0.15)	0.32	4.89
**Defending**					
6. Reciprocity	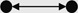	1.11	(0.28)**	0.61	12.01**
7. Transitivity	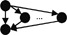	0.48	(0.13)**	0.24	1.82
8. Multiple two‐paths	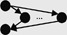	−0.11	(0.06)	0.14	10.71**
9. Shared in‐ties	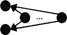	0.14	(0.21)	0.52	9.94**
**Victimization and defending**					
10. In‐ties aggression and defending	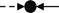	−0.03	(0.02)	0.04	15.95**
11. Out‐ties victimization and defending	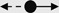	0.10	(0.06)	0.15	22.75**
12. In‐ties aggression and out‐ties defending	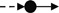	0.02	(0.01)	0.00	1.31
13. Out‐ties victimization and in‐ties defending	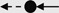	0.03	(0.05)	0.11	4.59
14. Defending for shared out‐ties of victimization	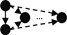	0.12	(0.07)	0.08	−0.10
15. Defending for shared in‐ties of aggression	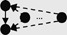	1.00	(0.26)**	0.41	2.05

*p < .05; **p < .01. The degree of freedom for the *χ* ^2^ test is 1. Dotted lines indicate victim‐aggressor relations, solid lines indicate defending relations in the graphical representations of the parameters. The mean parameter is an unstandardized aggregated estimate across classrooms. The standard deviation represents the degree to which estimates vary across classrooms. PE = parameter estimate; SE = standard error.

The second part of Table 2 contains effects for the defending network. Defending relations were likely to be *reciprocated* (#6; *P.E.=*1.11, *p *< .01) and *transitive* (i.e., there are defending triads: children defend the defenders of their defenders, #7; *P.E.=*0.48, *p *< .01). The strength of the *reciprocity, multiple two‐paths* (#8) and *shared in‐ties* (#9) parameters varied between classrooms. The latter two were included in the model to obtain good model fit.

The final part of Table 2 contains multivariate parameters with a combination of victimization and defending relations. On average, there was no association between receiving nominations for aggression and defending (#10), whereas there was a weak tendency that victimized children had defenders (#11; *P.E. = *0.10, *p *= .10). Testing H2, there was a small tendency that victims who shared aggressors defended each other (#14; *P.E. = *0.12, *p *= .06), and a strong tendency for defending among aggressors who targeted the same victims (#15; *P.E. = *1.00, *p *< .01).

Results for dominance and insecurity are given in Table 3. In line with H3, the likelihood for being nominated as an aggressor increased when children were socially dominant (#c2; *receiver* effect, *P.E. = *0.76, *p *< .01) and less insecure (#c5; *receiver* effect, *P.E. = *−0.21, *p *< .01). However, contrary to H4, dominance increased the likelihood for victimization relations (#c1; *sender* effect, *P.E. = *0.22, *p *< .01). It was not found that victims and their aggressors differed in dominance (#c3) or insecurity (#c6), which supports H5. In the defending network, victims had more defenders when they had lower levels of insecurity (#c10; *sender* effect, *P.E. = *−0.25, *p *< .01), suggesting that defended victims were less insecure than undefended victims. A relatively small negative difference effect for dominance was found (#c9; *P.E. = *−0.11, *p *< .01). The negative effect indicates that defenders and their victims were more similar in dominance than a random pair of children.

**Table 3:** Network models (ERGMs) for dominance and insecurity in victimization and defending

		Model 2: Dominance and insecurity			
		Mean parameter		Standard deviation	
Parameter	Graphical representation	PE	SE	Est.	*χ* ^2^
**Victimization**					
Dominance					
c1 Victim (sender)	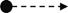	0.22	(0.07)**	0.13	1.81
c2. Aggressor (receiver)	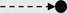	0.76	(0.17)**	0.43	29.50**
c3. Absolute difference	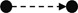	0.03	(0.14)	0.33	13.60
Insecurity					
c4. Victim (sender)	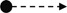	−0.06	(0.07)	0.10	0.75
c5. Aggressor (receiver)	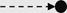	−0.21	(0.08)**	0.14	1.40
c6. Absolute difference	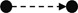	0.03	(0.07)	0.06	0.10
**Defending**					
Dominance					
c7. Victim (sender)	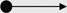	0.07	(0.11)	0.24	12.05**
c8. Defender (receiver)	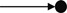	0.01	(0.06)	0.04	0.11
c9. Absolute difference	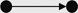	−0.11	(0.05)*	0.05	0.13
Insecurity					
c10. Victim (sender)	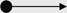	−0.25	(0.09)**	0.17	0.45
c11. Defender (receiver)	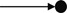	−0.16	(0.12)	0.26	6.22*
c12. Absolute difference	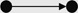	0.00	(0.11)	0.21	5.64*

*p < .05; **p < .01. The degree of freedom for the *χ* ^2^ test is 1. The mean parameter is an unstandardized aggregated estimate across classrooms. The standard deviation represents the degree to which estimates vary across classrooms.

The results for sex are given in Table 4. In line with H6a and H6b, boys were more aggressive than girls, whereas girls defended more than boys. Boy‐Boy (#s4; *P.E. = *0.93, *p < *.01) and Girl‐Boy (#s3; in which a girl is victimized by a boy; *P.E. = *0.79, *p < *.01) victimization dyads were more likely than Girl‐Girl (#s1) and Boy‐Girl dyads (#s2), partly confirming H7a. Defending was clearly a same‐sex phenomenon. In line with H7b, Boy‐Boy (#s8) defending relations were as likely as Girl‐Girl (#s5) defending relations, whereas it was less likely that boys were defended by girls (#s6; *P.E. = *−0.97, *p < *.01) or that girls were defended by boys (#s7; *P.E. = *−1.19, *p < *.01). Most sex effects had significant variation between classrooms (except for Girl‐Boy victimization).

**Table 4:** Network models (ERGMs) for sex in victimization and defending

		Model 3: Sex			
		Mean parameter		Standard deviation	
Parameter	Graphical representation	PE	SE	Est.	*χ* ^2^
**Victimization**					
s1. Girl‐Girl^a^	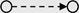				
s2. Boy‐Girl	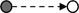	−0.32	(0.29)	0.69	16.55**
s3. Girl‐Boy	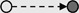	0.79	(0.20)**	0.43	3.63
s4. Boy‐Boy	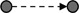	0.93	(0.23)**	0.54	9.94**
**Defending**					
s5. Girl‐Girl^b^	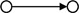				
s6. Boy‐Girl	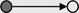	−0.97	(0.42)**	1.04	53.38**
s7. Girl‐Boy	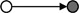	−1.19	(0.36)**	0.87	32.18**
s8, Boy‐Boy	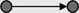	−0.19	(0.27)	0.63	16.93**

*p < .05; **p < .01. The degree of freedom for the *χ* ^2^ test is 1. The mean parameter is an unstandardized aggregated estimate across classrooms. The standard deviation represents the degree to which estimates vary across classrooms.

^a^ The first person in the dyad is the sender (victim), the second person is the receiver (aggressor); that is, Boy‐Girl means that a boy is victimized by a girl.

^b^ The first person in the dyad is the sender (defended victim), the second person is the receiver (defender)

Table 5 gives the full multivariate model that combines structural parameters for victimization and defending, and effects for dominance, insecurity, and sex. For the parameters in the victimization part, some estimates changed considerably when compared with the univariate analyses. The effects of the *in‐ties spread* (#2), sex (aggression by boys, #s3 and #s4), and the receiver effect for dominance (#c2) reduced in strength. This can be explained by their strong overlap. Boys were often nominated for aggression, and dominance correlated strongly with receiving nominations for aggression (*r *= .48, *p *< .01, see Appendix 1 for all correlations). In separately estimated models, it was indeed found that the inclusion of structural network parameters for aggression reduced the strength of effects both for sex and dominance/insecurity (see Appendices for these extra analyses). For the defending part, inclusion of structural parameters did not affect the parameter estimates for dominance/insecurity and sex substantively (compare the estimates in Table 5 with the estimates in Tables 2, 3, 4). Finally, the multivariate victimization‐defending parameters (#10–#15) did not change considerably in the full model when compared with Table 1.

**Table 5:** Multivariate network models (ERGMs) for victimization and defending, sex, and dominance and insecurity

		Model 4: Full model			
		Mean parameter		Standard deviation	
Parameter	Graphical representation	PE	SE	Est.	*χ* ^2^
**Victimization**					
1. Reciprocity	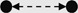	0.24	(0.16)	0.00	0.00
2. In‐ties spread	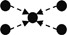	−0.02	(0.41)	0.85	2.99
3. Multiple two‐paths	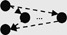	−0.02	(0.03)	0.06	1.98
4. Shared in‐ties	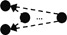	−0.29	(0.11)*	0.07	4.86
5. Shared out‐ties	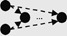	0.22	(0.16)	0.35	5.87*
Relational covariates					
s1. Girl‐Girl	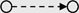				
s2. Boy‐Girl	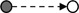	−0.71	(0.37)	0.83	9.59**
s3. Girl‐Boy	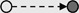	−0.25	(0.28)	0.66	21.80**
s4. Boy‐Boy	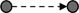	−0.20	(0.15)	0.00	0.00
Dominance					
c1 Victim (sender)	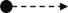	0.34	(0.17)*	0.38	13.16**
c2. Aggressor (receiver)	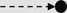	0.19	(0.04)**	0.00	0.00
c3. Absolute difference	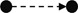	0.02	(0.09)	0.18	4.02
Insecurity					
c4. Victim (sender)	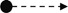	0.12	(0.16)	0.33	7.22*
c5. Aggressor (receiver)	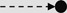	−0.07	(0.04)	0.03	0.05
c6. Absolute difference	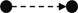	0.01	(0.05)	0.00	0.00
**Defending**					
6. Reciprocity	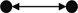	0.88	(0.22)**	0.42	4.78
7. Transitivity	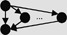	0.34	(0.13)**	0.25	3.78
8. Multiple two‐paths	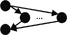	−0.06	(0.05)	0.10	1.69
9. Shared in‐ties	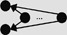	0.25	(0.33)	0.84	18.07**
Relational covariates					
s5. Girl‐Girl	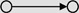				
s6. Boy‐Girl	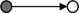	−0.98	(0.44)*	1.07	31.85**
s7. Girl‐Boy	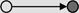	−1.10	(0.23)**	0.45	2.38
s8, Boy‐Boy	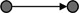	−0.17	(0.13)	0.00	0.00
Dominance					
c7. Victim (sender)	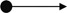	−0.06	(0.09)	0.16	2.32
c8. Defender (receiver)	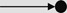	−0.09	(0.06)	0.00	0.00
c9. Absolute difference	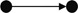	−0.09	(0.05)	0.03	0.03
Insecurity					
c10. Victim (sender)	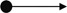	−0.14	(0.10)	0.20	6.15*
c11. Defender (receiver)	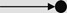	−0.17	(0.10)	0.18	3.77
c12. Absolute difference	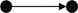	0.00	(0.09)	0.16	4.07
**Victimization and defending**					
10. In‐ties aggression and defending	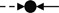	−0.01	(0.02)	0.04	11.08**
11. Out‐ties victimization and defending	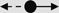	0.13	(0.05)**	0.10	5.44*
12. In‐ties aggression and out‐ties defending	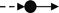	0.01	(0.01)	0.03	3.91
13. Out‐ties victimization and in‐ties defending	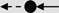	0.07	(0.06)	0.13	13.72**
14. Defending for shared out‐ties of victimization	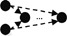	0.08	(0.06)	0.00	0.00
15. Defending for shared in‐ties of aggression	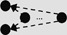	0.82	(0.21)**	0.29	1.12

*p < .05; **p < .01. The degree of freedom for the *χ* ^2^ test is 1. Dotted lines indicate victim‐aggressor relations, solid lines indicate defending relations in the graphical representations of the parameters. The mean parameter is an unstandardized aggregated estimate across classrooms. The standard deviation represents the degree to which estimates vary across classrooms.

## 4 DISCUSSION

The current study employed social network analysis to examine peer victimization, aggression, and defending relationships simultaneously during early childhood. We examined these relationships within the same classroom, accounting for children's sex, dominance, and insecurity. These analyses were framed to examine whether young aggressors are less selective in their choice of targets. It is vital to understand more about these social processes involving the wider peer‐group in early childhood, which may be particularly beneficial for prevention and intervention programs among this age group (Barker et al., 2008).

### 4.1 Selectivity in victimization and aggression relations

Several of the findings indicate that aggressors in early childhood are less selective in their choice of suitable, vulnerable targets than aggressors in late childhood or adolescence. In support of hypothesis 1, there was reciprocity in aggression, meaning that some children were mutually aggressive. Thus, aggression in early childhood is often not unidirectional, which may be more a characteristic of behavior identified in later childhood such as bullying (Smith, 2011). Moreover, in the victimization/aggression network, some children clearly received more nominations for aggression than others. This indicates that aggressive children have a reputation of being aggressive among their peers, and it also suggests that young children are able to discriminate their nominations for aggression.

This research enabled the unique examination of the multivariate links between victimization and defending ties which serve affection needs. With hypothesis 2, we expected that defending among aggressors would be more likely than defending among victims, because aggression might be a more stable behavioral pattern in early childhood than victimization. We found that defending among aggressors sharing victims (see Figure 1a) was more likely than defending among victims targeted by the same aggressors (see Figure 1b). This suggests that aggressive children in early childhood are already able to support each other and provide affection, even though the supportive aggressive roles of assistant and reinforcer are not clearly defined at this age (Camodeca et al., 2015; Monks & Smith, 2010; Monks et al., 2003). The relative lack of defending among young victims supports the explanation that aggressors are less selective, which implies that victimization would be less likely an enduring experience for most children. This makes it more difficult for victims to identify others with whom they share their plight and satisfy affection needs (Kochenderfer‐Ladd & Wardrop, 2001; Monks et al., 2003; Persson, 2005; Snyder et al., 2003).

More support for the lack of selectivity in aggressors’ target choice was found in children's social dominance and insecurity characteristics. The findings supported hypothesis 3: aggression was associated with high dominance and low insecurity (Hawley & Geldhof, 2012; Pellegrini et al., 2007; Perren & Alsaker, 2006). In contrast to hypothesis 4, however, victimization was associated with dominance (and unrelated to insecurity). These findings contrast with studies with older children (Olthof, Goossens, Vermande, Aleva, & Van der Meulen, 2011; Scholte, Engels, Overbeek, de Kemp, & Haselager, 2007) and some of the research with younger victims (Perren & Alsaker, 2006; Schwartz, Dodge, & Coie, 1993). The current findings align with the proposition that victimization in early childhood is a transient experience for many, and that aggressors do not strategically target the most vulnerable victims. In fact, aggressors may target children who are dominant and aggressive themselves, possibly to compete for a good position in the hierarchy, which increases the chances for retaliation. The finding that victims did not differ in dominance and insecurity from their specific aggressors also aligns with this, and supports hypothesis 5. It is possible that children who are lower in dominance or more insecure may be those who are later targeted for repeated victimization, or that current experiences may further negatively impact on their dominance and feelings of security. The lack of selectivity is relevant for early childhood interventions, because systematic victimization by selective bullies can have severe mental health consequences (e.g., Arseneault, 2018).

Also, children's sex was not found to be a selective factor that limits children's social interaction patterns in terms of choice of target of aggression. Boys were found to be more aggressive than girls (hypothesis 6a). However, contrary to hypothesis 7a, stating that same‐sex aggression would be more common than cross‐sex aggression, we found that boys were aggressive to both boys and girls. Also girls were equally likely to be aggressive to both girls and boys. These results are in line with earlier research and indicate that young children are less strategic than older children in choosing targets and are being aggressive to both cross‐sex and same‐sex peers (see Veenstra et al., 2013). Furthermore, the relative difference in cross‐sex aggression observed in the current study, with boys being more likely to be identified as behaving aggressively toward girls than vice versa, is in contrast with studies among older age groups. Research with older children and adolescents has consistently reported that girls physically aggress more to boys than boys physically victimize girls (Archer, 2004). This difference in findings may be reflective of age differences in patterns of aggression or the composite measure of aggression that we used.

### 4.2 Defending networks in early childhood

Sex significantly explained the construction of defending relations. Girls were more likely than boys to be identified as defenders in line with hypothesis 6b and previous research (Belacchi & Farina, 2010; Lee et al., 2016; Monks et al., 2011, 2003) and defending was clearly same‐sex, in line with hypothesis 7b. Defending starts to play an important role in the affection needs of young children because same‐sex classmates are the most important ingroup at this age (Veenstra et al., 2013).

The defending networks were further characterized by reciprocity, meaning that children mutually defended each other, and transitivity, meaning that children defended the defenders of their defenders. Reciprocity and transitivity are the building blocks for larger cohesive groups (e.g., Schaefer, Light, Fabes, Hanish, & Martin, 2010). This suggests that defending relations appear within relatively close subgroups of children. Children may defend their friends at this age (Sainio et al., 2011), which may also account for more frequent same‐sex defending. These findings would lend support to the hypothesis that young children may be using defending somewhat selectively as proposed by Veenstra et al. (2010).

A result not predicted was that defended victims were less insecure than undefended victims, supporting previous findings that defended victims are better adjusted (Sainio et al., 2011). Children who are less insecure may be better able to have good relations with peers, meaning that they are more likely to be defended. A causally reversed explanation is that defending might contribute to increased feelings of security. Dominance did not further qualify defending relations, except for a small negative difference effect, suggesting that victims and their defenders were somewhat more similar than another pair of children.

### 4.3 Limitations, strengths, and implications

In this research, we relied on peer reports of victim‐aggressor and victim‐defending relations. Although even young peers can serve as valuable informants for behavior of classmates, we were not able to account for children's own victimization experiences. It might be that children at this young age have difficulties in accurately imagining the feelings and experiences of peers. Another limitation is that this research was conducted at one point in time, thereby precluding statements about developmental social processes. It would be of interest to examine how victimization, aggression, and defending behavior changes and develops across the formative school years. To date, there is little research that has employed a longitudinal social network approach to peer victimization and defending, and none has examined how these simultaneously develop and change during the early school years.

We made use of a unique dataset with an initial sample size of 200 children, who were all individually interviewed, from eight classrooms (of which we were able to estimate seven in our network models). Despite the relatively small sample, the findings of the meta‐analysis on the network models indicate that there are small differences between the classrooms in terms of the size of the estimated effect for the parameters (as indicated by the standard deviation), especially for the parameters that investigate the interplay between victimization/aggression and defending.

This research has indicated that it is vital to take a social network approach to understanding the intertwined relational structures of peer victimization and defending among young children. The findings indicate that early childhood aggressors are less selective in choosing vulnerable targets, but aggressors do defend each other. This implies that young aggressive children already form alliances with other aggressive children, and suggests that interventions should focus on social processes early on in children's schooling. Therefore, it might be useful to address this in interventions by facilitating children's interaction with many classmates to provide young children with the opportunity to observe and experience behavioral alternatives. Moreover, it is possible that young aggressors defend each other to prevent retaliation by victims or from defenders of the victims (e.g., Huitsing et al., 2014). The support between aggressive children may act as a form of reinforcement for this behavior. Effective interventions in early childhood could additionally focus on peer reinforcement of aggression, which may be beneficial in reducing peer victimization among young children.

## CONFLICTS OF INTEREST

The authors report no conflict of interest.

## Supporting information

Additional Supporting Information may be found online in the supporting information tab for this article.

**Appendix S1**. Descriptives of: Mean, Standard Deviations, and correlations among received and given nominations, sex, and dominance and insecurity.
**Appendix S2**. Multivariate Network Models (ERGMs) for Victimization and Defending and Sex.
**Appendix S3**. Multivariate Network Models (ERGMs) for Victimization and Defending, and Dominance and Insecurity.
**Appendix S4**. Multivariate Network Models (ERGMs) for Sex and Dominance and Insecurity in Victimization and Defending.
