# Supplementary material for: Who victimizes whom and who defends whom? A multivariate social network analysis of victimization, aggression, and defending in early childhood
Source: Aggress Behav. 2018 Mar 25;44(4):394–405. doi: 10.1002/ab.21760 (PMC6033031; doi:10.1002/ab.21760)
Supplement: Supplementary file 1 — Appendix S1. Descriptives of: Mean, Standard Deviations, and correlations among received and given nominations, sex, and dominance and insecurity. Appendix S2. Multivariate Network Models (ERGMs) for Victimization and Defending and Sex. Appendix S3. Multivariate Network Models (ERGMs) for Victimization and Defending, and Dominance and Insecurity. Appendix S4. Multivariate Network Models (ERGMs) for Sex and Dominance and Insecurity in Victimization and Defending. [file AB-44-394-s001.docx]

**Appendices (online only available)**

The tables in the Appendix were given to provide more insights into the interrelatedness between the parameter estimates in the network models.

**Appendix 1** provides an overview of the means, standard deviations, and correlations among the study variables. For this table we made use of the proportion of nominations, in which the number of nominations given/received was divided by the total number of nominators in the classroom. This yielded scores from 0 (not nominated at all) to 1 (nominated by all classmates). Correlating these proportions with the child characteristics provide some preliminary insights into their relatedness, without accounting for the complex overall network structure and the nesting of these relations within children.

The correlation table shows that involvement in victimization, aggression, and defending were all related. For example, aggression was related to victimization (*r*=.42, *p*<.01) and being defended was related to defending others (*r*=.69, *p*<.01). Girls were less likely to be aggressors (*r*=-.31, *p*<.01) and more likely to be defenders (*r*=.17, *p*<.05) than boys. Teacher-rated dominance was related to aggression (*r*=.48, *p*<.01), victimization (*r*=.37, *p*<.01), defending others (*r*=.35, *p*<.01), and being defended (*r*=.42, *p*<.01). Insecurity was negatively related with dominance (*r*=.-22, *p*<.01). Moreover, insecurity made it less likely to be mentioned for being aggressive (*r*=-.18, *p*<.01).

**Appendices 2-4** were included to demonstrate with a stepwise procedure how the parameter estimates in the network models change under the influence of the combination of a cluster of parameters (i.e., structural parameters, parameters for dominance/insecurity, sex). Note that the network models with sex or dominance/insecurity only (thus, without structural network parameters) account for the relational nature of the data and the nesting of relations within children.

The combination of the models with structural parameters (model 1) and sex (model 3) can be found in **Appendix 2**. Compared with the parameter estimates in model 1, the estimate for the *in-ties spread* of aggression dropped after the inclusion of sex. Similarly, compared with model 3, the parameter estimates for sex significantly changed after the inclusion of structural parameters. This can be explained by the finding that the children who were frequently nominated for aggression (received nominations, modelled by the *in-ties spread*) were most often boys; there were 41 children who were aggressive towards more than 50% of their classmates; 34 of these (83%) were boys. The parameter estimates for the defending network and the multivariate victimization-defending combination did not change substantively when models 1 and 3 were combined into Model A1.

**Appendix 3** provides model A2, a combination of models 1 (structural parameters) and 2 (dominance and insecurity). Compared with model 1, the estimates for the *reciprocity* parameter and the *in-ties spread* for aggression dropped significantly. Similarly, compared with model 2, the estimate for the receiver effect for *aggressor* strongly dropped after the inclusion of structural parameters. The explanation can be found in the strong association between dominance and both aggression and victimization, as already shown by the correlations with the proportion of nominations (*r*s=.48 and .37, respectively). Although dominance was also found to be related to defending others and being defended (*r*s=.35 and .42, respectively), including sender, receiver, and difference parameters for dominance did not alter substantively the estimates for the structural parameters for defending. This suggest that the defending network, in comparison with the victimization/aggression network, has a more complex structure that cannot fully be explained by characteristics at the child level.

Sex was uncorrelated with dominance (*r*=.00) and insecurity (*r*=.04). The combination of models 2 (dominance and insecurity) and 3 (sex) in **Appendix 4** show, in line with these correlations, that the parameters estimates of Models 2 and 3 remain relatively stable in the combined model A3.

**Appendix 1 (available online)**: Descriptives of: Mean, Standard Deviations, and correlations among received and given nominations, sex, and dominance and insecurity.

|  | M | SD |  | Correlations | | | | | |
| --- | --- | --- | --- | --- | --- | --- | --- | --- | --- |
|  |  |  |  | 1. | 2. | 3. | 4. | 5. | 6. |
| 1. Aggressor (Victimization received) | 0.29 | (0.29) |  |  |  |  |  |  |  |
| 1. Victim (Victimization given) | 0.29 | (0.14) |  | .42** |  |  |  |  |  |
| 1. Defender (Defending received) | 0.30 | (0.21) |  | .15* | .46** |  |  |  |  |
| 1. Defended victim (Defending given) | 0.30 | (0.23) |  | .33** | .49** | .69** |  |  |  |
| 1. Sex (1=Girl) | 0.46 | - |  | -.31** | .02 | .17* | .04 |  |  |
| 1. Dominance | 2.51 | (1.08) |  | .48** | .37** | .35** | .42** | .00 |  |
| 1. Insecurity | 2.28 | (1.03) |  | -.18* | -.04 | .05 | -.04 | .04 | -.22** |

*Note.* **P<*.05; ***P<*.01.

**Appendix 2 (available online)**: Multivariate Network Models (ERGMs) for Victimization and Defending and Sex

| **Parameter** | **Graphical**  **representation** | **Model A1:  Combination of Models 1 and 3 of the Manuscript** | | | | |
| --- | --- | --- | --- | --- | --- | --- |
|  |  | Mean parameter | |  | Standard deviation | |
|  |  | PE | SE |  | Est. | χ^2^ |
| **Victimization** |  |  |  |  |  |  |
| 1. Reciprocity |  | 0.50 | (0.18)** |  | 0.25 | 0.08 |
| 2. In-ties spread |  | 0.36 | (0.35) |  | 0.66 | 0.26 |
| 3. Multiple two-paths | … | 0.00 | (0.03) |  | 0.07 | 1.31 |
| 4. Shared in-ties | … | -0.24 | (0.11)* |  | 0.22 | 2.78 |
| 5. Shared out-ties | … | 0.30 | (0.16) |  | 0.33 | 4.72 |
| *Relational covariates* |  |  |  |  |  |  |
| s1. Girl-Girl |  |  |  |  |  |  |
| s2. Boy-Girl |  | -0.71 | (0.38) |  | 0.88 | 8.62** |
| s3. Girl-Boy |  | -0.26 | (0.28) |  | 0.68 | 22.84** |
| s4. Boy-Boy |  | -0.32 | (0.14)* |  | 0.00 | 0.00 |
| **Defending** |  |  |  |  |  |  |
| 6. Reciprocity |  | 0.92 | (0.22)** |  | 0.43 | 3.53 |
| 7. Transitivity | … | 0.41 | (0.11)** |  | 0.19 | 0.07 |
| 8. Multiple two-paths | … | -0.05 | (0.06) |  | 0.13 | 3.90 |
| 9. Shared in-ties | … | 0.28 | (0.37) |  | 0.96 | 13.90** |
| *Relational covariates* |  |  |  |  |  |  |
| s5. Girl-Girl |  |  |  |  |  |  |
| s6. Boy-Girl |  | -0.90 | (0.46)** |  | 1.12 | 33.87** |
| s7. Girl-Boy |  | -1.00 | (0.27)** |  | 0.59 | 3.84 |
| s8, Boy-Boy |  | -0.07 | (0.12) |  | 0.12 | -0.10 |
| **Victimization and defending** |  |  |  |  |  |  |
| 10. In-ties aggression and defending |  | -0.02 | (0.02) |  | 0.04 | 9.48** |
| 11. Out-ties victimization and defending |  | 0.13 | (0.06)* |  | 0.14 | 15.80** |

*Table continues on next page.*

**Appendix 2 (available online)**. Continued.

| 12. In-ties aggression and out-ties defending |  | 0.02 | (0.01) |  | 0.00 | 1.39 |
| --- | --- | --- | --- | --- | --- | --- |
| 13. Out-ties victimization and in-ties defending |  | 0.06 | (0.07) |  | 0.16 | 16.59** |
| 14. Defending for shared out-ties of victimization | … | 0.08 | (0.06) |  | 0.00 | 0.00 |
| 15. Defending for shared in-ties of aggression | … | 0.92 | (0.25)** |  | 0.38 | 1.17 |

*Note.* **P<*.05; ***P<*.01. The degree of freedom for the χ^2^ test is 1. Dotted lines indicate victim-aggressor relations, solid lines indicate defending relations in the graphical representations of the parameters. The mean parameter is an unstandardized aggregated estimate across classrooms. The standard deviation represents the degree to which estimates vary across classrooms.

**Appendix 3 (available online)**: Multivariate Network Models (ERGMs) for Victimization and Defending, and Dominance and Insecurity

| **Parameter** | **Graphical**  **representation** | **Model A2:  Combination of Models 1 and 2 of the Manuscript** | | | | |
| --- | --- | --- | --- | --- | --- | --- |
|  |  | Mean parameter | |  | Standard deviation | |
|  |  | PE | SE |  | Est. | χ^2^ |
| **Victimization** |  |  |  |  |  |  |
| 1. Reciprocity |  | 0.30 | (0.16) |  | 0.00 | 0.00 |
| 2. In-ties spread |  | 0.17 | (0.35) |  | 0.63 | 0.52 |
| 3. Multiple two-paths | … | -0.05 | (0.04) |  | 0.08 | 2.35 |
| 4. Shared in-ties | … | -0.24 | (0.09)** |  | 0.09 | 0.13 |
| 5. Shared out-ties | … | 0.19 | (0.15) |  | 0.32 | 7.70** |
| *Dominance* |  |  |  |  |  |  |
| c1 Victim (sender) |  | 0.31 | (0.16) |  | 0.37 | 15.14** |
| c2. Aggressor (receiver) |  | 0.16 | (0.03)** |  | 0.00 | 0.00 |
| c3. Absolute difference |  | 0.01 | (0.08) |  | 0.18 | 12.72** |
| *Insecurity* |  |  |  |  |  |  |
| c4. Victim (sender) |  | 0.14 | (0.150 |  | 0.33 | 7.50* |
| c5. Aggressor (receiver) |  | -0.05 | (0.04) |  | 0.05 | 0.30 |
| c6. Absolute difference |  | 0.01 | (0.05) |  | 0.00 | 0.00 |
| **Defending** |  |  |  |  |  |  |
| 6. Reciprocity |  | 1.08 | (0.27)** |  | 0.61 | 16.04** |
| 7. Transitivity | … | 0.39 | (0.14)** |  | 0.26 | 3.76 |
| 8. Multiple two-paths | … | -0.12 | (0.05)* |  | 0.09 | 3.74 |
| 9. Shared in-ties | … | 0.10 | (0.18) |  | 0.45 | 9.14* |
| *Dominance* |  |  |  |  |  |  |
| c7. Victim (sender) |  | -0.03 | (0.10) |  | 0.19 | 6.05* |
| c8. Defender (receiver) |  | -0.05 | (0.06) |  | 0.00 | 0.00 |
| c9. Absolute difference |  | -0.06 | (0.04) |  | 0.00 | 0.00 |
| *Insecurity* |  |  |  |  |  |  |
| c10. Victim (sender) |  | -0.13 | (0.09) |  | 0.16 | 4.34 |
| c11. Defender (receiver) |  | -0.16 | (0.09) |  | 0.16 | 2.85 |
| c12. Absolute difference |  | 0.01 | (0.07) |  | 0.12 | 2.10 |

*Table continues on next page.*

**Appendix 3 (available online)**. Continued.

| **Victimization and defending** |  |  |  |  |  |  |
| --- | --- | --- | --- | --- | --- | --- |
| 10. In-ties aggression and defending |  | -0.03 | (0.02) |  | 0.04 | 22.62** |
| 11. Out-ties victimization and defending |  | 0.11 | (0.05)* |  | 0.12 | 16.38** |
| 12. In-ties aggression and out-ties defending |  | 0.02 | (0.01) |  | 0.01 | 4.34 |
| 13. Out-ties victimization and in-ties defending |  | 0.04 | (0.04) |  | 0.08 | 3.73 |
| 14. Defending for shared out-ties of victimization | … | 0.12 | (0.06)* |  | 0.03 | 0.01 |
| 15. Defending for shared in-ties of aggression | … | 0.88 | (0.23)** |  | 0.34 | 2.34 |

*Note.* **P<*.05; ***P<*.01. The degree of freedom for the χ^2^ test is 1. Dotted lines indicate victim-aggressor relations, solid lines indicate defending relations in the graphical representations of the parameters. The mean parameter is an unstandardized aggregated estimate across classrooms. The standard deviation represents the degree to which estimates vary across classrooms.

**Appendix 4 (available online)**: Multivariate Network Models (ERGMs) for Sex and Dominance and Insecurity in Victimization and Defending,

| **Parameter** | **Graphical**  **representation** | **Model A3:  Combination of Models 2 and 3 of the Manuscript** | | | | |
| --- | --- | --- | --- | --- | --- | --- |
|  |  |  | |  | Standard deviation | |
|  |  | PE | SE |  | Est. | χ^2^ |
| **Victimization** |  |  |  |  |  |  |
| *Relational covariates* |  |  |  |  |  |  |
| s1. Girl-Girl |  |  |  |  |  |  |
| s2. Boy-Girl |  | -0.41 | (0.30) |  | 0.68 | 16.46** |
| s3. Girl-Boy |  | 0.83 | (0.28)** |  | 0.65 | 16.31** |
| s4. Boy-Boy |  | 0.97 | (0.29)** |  | 0.68 | 21.24** |
| *Dominance* |  |  |  |  |  |  |
| c1 Victim (sender) |  | 0.24 | (0.07)** |  | 0.11 | 1.50 |
| c2. Aggressor (receiver) |  | 0.78 | (0.16)** |  | 0.40 | 22.14** |
| c3. Absolute difference |  | 0.03 | (0.13) |  | 0.30 | 13.18** |
| *Insecurity* |  |  |  |  |  |  |
| c4. Victim (sender) |  | -0.07 | (0.06) |  | 0.06 | 0.17 |
| c5. Aggressor (receiver) |  | -0.24 | (0.12)* |  | 0.24 | 9.29** |
| c6. Absolute difference |  | 0.02 | (0.06) |  | 0.00 | 0.00 |
| **Defending** |  |  |  |  |  |  |
| *Relational covariates* |  |  |  |  |  |  |
| s5. Girl-Girl |  |  |  |  |  |  |
| s6. Boy-Girl |  | -1.06 | (0.38)** |  | 0.92 | 41.95** |
| s7. Girl-Boy |  | -1.27 | (0.34)** |  | 0.81 | 27.73** |
| s8, Boy-Boy |  | -0.28 | (0.24) |  | 0.54 | 12.17** |
| *Dominance* |  |  |  |  |  |  |
| c7. Victim (sender) |  | 0.04 | (0.10) |  | 0.21 | 9.22** |
| c8. Defender (receiver) |  | 0.01 | (0.05) |  | 0.00 | 0.00 |
| c9. Absolute difference |  | -0.12 | (0.06) |  | 0.09 | 0.49 |
| *Insecurity* |  |  |  |  |  |  |
| c10. Victim (sender) |  | -0.26 | (0.06)** |  | 0.04 | 0.01 |
| c11. Defender (receiver) |  | -0.16 | (0.11) |  | 0.23 | 4.53 |
| c12. Absolute difference |  | 0.00 | (0.11) |  | 0.20 | 5.81* |

*Note.* **P<*.05; ***P<*.01. The degree of freedom for the χ^2^ test is 1. The mean parameter is an unstandardized aggregated estimate across classrooms. The standard deviation represents the degree to which estimates vary across classrooms.
